# Analysis of the Development Trajectory and Influencing Factors of Depression in Patients With Cervical Cancer During Concurrent Chemoradiotherapy

**DOI:** 10.62641/aep.v53i6.2085

**Published:** 2025-12-17

**Authors:** Qing He, Lun Xiao, Ying Lan, Desheng Yao

**Affiliations:** ^1^Department of Radiation Oncology, Guangxi Medical University Cancer Hospital, 530021 Nanning, Guangxi , China; ^2^Department of Urology, The First Affiliated Hospital of Guangxi Medical University, 530021 Nanning, Guangxi, China; ^3^Department of Gynecologic Oncology, Guangxi Medical University Cancer Hospital, 530021 Nanning, Guangxi, China

**Keywords:** cervical cancer, concurrent chemoradiotherapy, depression

## Abstract

**Background:**

This study aims to analyse the developmental trajectory of depression in patients undergoing concurrent chemoradiotherapy (CCRT) for cervical cancer and its influencing factors.

**Methods:**

A retrospective analysis of clinical data was performed on 160 patients with cervical cancer who received CCRT at our hospital between July 2023 and June 2025. Individuals with depression were assigned to the depressed group, whereas those without depression were assigned to the non-depressed group. Employing latent class growth modelling to identify depression trajectories in cervical cancer patients undergoing CCRT. The factors influencing the latent classes of depression trajectories in patients were analysed through logistic regression.

**Results:**

The depressed group had higher rates of household monthly income per capita of less than 5000 RMB (1 USD = 7.1 RMB), stage III/IV tumour stage and avoidance/submission coping methods than the non depressed group (*p* = 0.001, 0.030, < 0.001) and had lower Multidimensional Scale of Perceived Social Support (MSPSS) scores (*p* = 0.001). Three distinct depression trajectories were identified: a low-level stable group (n = 31), a moderate-level increasing group (n = 54) and a high-level decreasing group (n = 29). The logistic regression analysis results indicated that patients with a household income per capita below 5000 RMB, stage III/IV tumour stage, avoidance/submission coping style and lower MSPSS scores exhibited a higher likelihood of entering the medium-level rising group and the high-level declining group compared to the other group (*p* < 0.05).

**Conclusions:**

Depression in patients with cervical cancer exhibits three distinct developmental trajectories. Household income per capita, tumour stage, coping style and MSPSS score may influence these trajectories. Thus, prompt intervention targeting these potential influencing factors is essential for managing the progression of depression.

## Introduction

Cervical cancer is a malignant tumour with a high morbidity and mortality rate. It is one of the most common cancer types affecting the female reproductive system [1]. As the condition advances, the tumour in the cervix will eventually infiltrate the surrounding normal tissues and may potentially spread to lymph nodes, posing a major threat to the patient’s life [2]. Current clinical approaches for treating cervical cancer include surgical resection, simple radiotherapy, adjuvant chemotherapy and concurrent chemoradiotherapy (CCRT) [3]. CCRT, which is a critical therapeutic option for cervical cancer, has been shown to improve patients’ pathology indicators, increase treatment efficiency and lower local recurrence rates. However, this therapy increases the probability of patients experiencing symptoms, such as loss of appetite, nausea, vomiting, depression and fatigue [4]. Patients with cervical cancer are more likely to experience sadness [5]. High levels of depression in patients with cervical cancer and on CCRT were strongly linked to reduced quality of life, cognitive function and social interaction abilities [6]. Long-term depression not only can make patients miss optimal therapy opportunities and lengthen the treatment cycle but also can impair treatment adherence [7]. Therefore, effective intervention measures are urgently required to ameliorate the depression of cervical cancer patients undergoing CCRT [8]. The latent growth mixture model (LGMM) identifies developmental trajectories for specific events across patient groups while accounting for individual differences, exceeding classic growth curve models in capturing event-specific heterogeneity [9]. As a result, the purpose of this study is to use the LGMM to analyse the development trajectory of depression in cervical cancer patients receiving CCRT, as well as to investigate the factors that influence it, in order to provide personalised intervention strategies for depression management by identifying different depression trajectory.

## Material and Methods

### Research Subjects

**Fig. 1.**
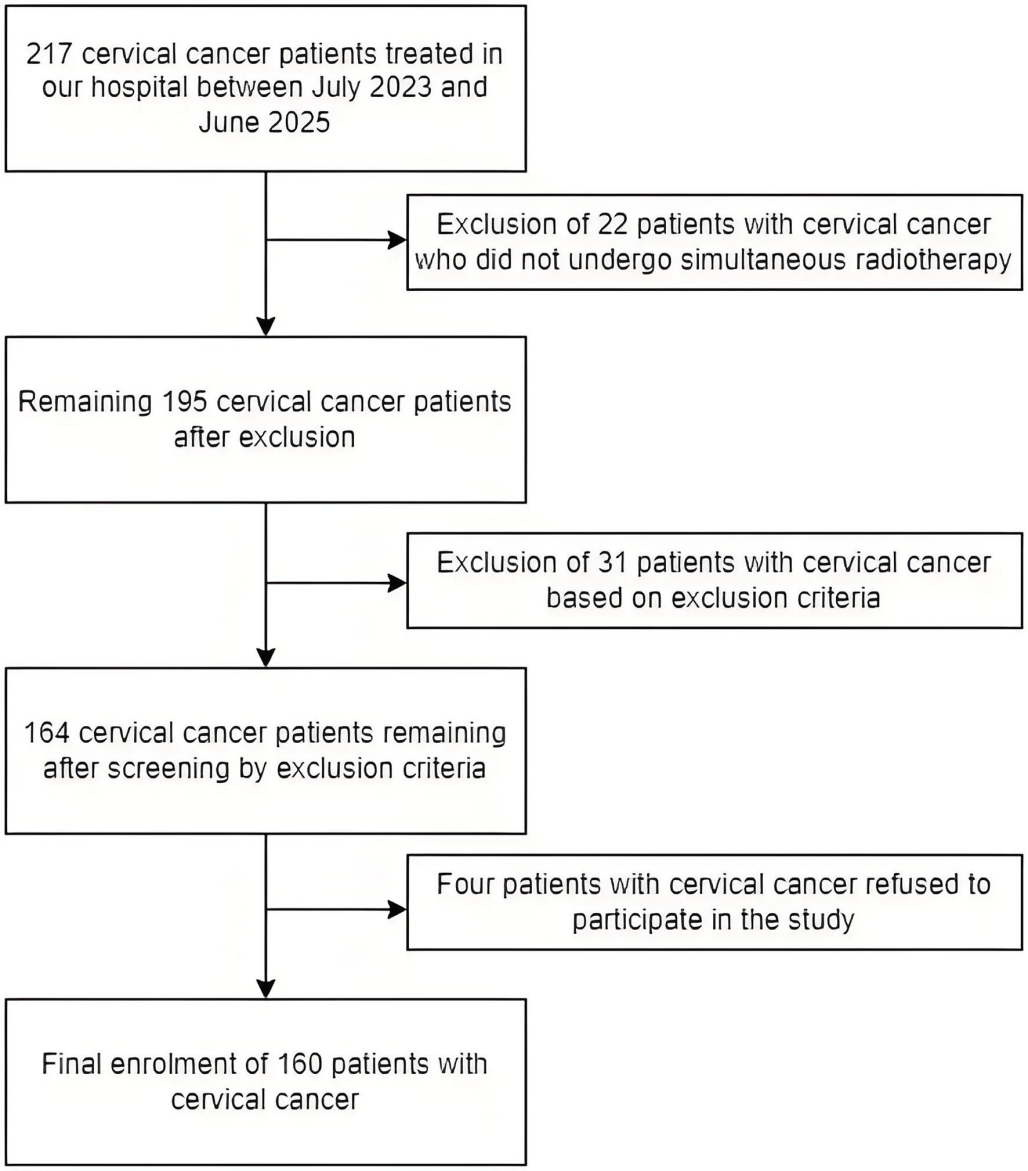
**Flowchart of screening for patients with cervical cancer**.

A retrospective review of the clinical data of 160 cervical cancer patients who had CCRT at our hospital from July 2023 to June 2025 was performed. Patients were separated into two groups based on whether they were depressed or not. The inclusion criteria were as follows: age of ≥18 years; pathological examination confirming diagnosis of locally advanced cervical cancer on the basis of clinical staging criteria (FIGO 2018 Edition) [10]; treatment with CCRT; No prior radiotherapy, chemotherapy or biological therapy; estimated survival of at least 3 months according to historical clinical evaluation; complete clinical data; voluntary participation and signed informed consent. The following exclusion criteria were used: concurrent infection, haematologic, endocrine or immune system disease; pregnancy or lactation; neuropsychiatric problems inhibiting participation; other malignancies; History of psychiatric illness or alcohol dependence/abuse. Previous use of antianxiety, depressive or sedative drugs; Distant metastases. Fig. 1 shows the patient screening process in detail. A total of 217 cervical cancer patients were screened, of which 22 were excluded because they did not undergo simultaneous radiotherapy, 7 were excluded because they had an expected survival of less than 3 months, 15 were excluded because they had been using anxiolytic, antidepressant or sedative drugs, 9 were excluded because of the presence of haematological disorders and 4 were excluded because they refused to participate in the study and 160 were eventually included in This study meets the necessary requirements of the World Medical Association’s Helsinki Declaration. This study was reviewed by the Science and Technology Ethics Committee of the Affiliated Tumor Hospital of Guangxi Medical University (No. KY20251016).

### Survey Tools

#### General Information Questionnaire

The patients’ age, body mass index (BMI), education level, marital status, monthly household income, menopause, Human papilloma virus (HPV) infection, tumour stage, lymph node metastasis, coping style, social support and squamous cell carcinoma antigen (SCC-Ag) level were collected.

#### Hospital Anxiety and Depression Scale

The depression subscale of the Hospital Anxiety and Depression Scale [11] was used in the assessment of patients’ levels of depression. It comprises of seven items, each of which is evaluated on a four-point scale from 0 to 3. The overall score is 0–21 points. The level of depression increases with score. Scores of 8–21 indicate depression (mild depression = 8–10 points; moderate depression = 11–14; severe depression = 15–21). The Cronbach’s α coefficient for the depression subscale was 0.750.

#### Medical Coping Style Scale

The Medical Coping Modes Questionnaire (MCMQ) was used to examine individuals’ coping strategies with disease [12]. It has three dimensions: facing (eight items), avoiding (seven items) and yielding (five items). The MCMQ has a four-point scoring system: 1, “never”; 2, “sometimes”; 3, “often”; and 4, “always”. The higher the score on all 20 elements, the more likely the patient is to use this coping method. The overall Cronbach’s α coefficient for this scale was 0.815, indicating good reliability.

#### Perceived Social Support Scale

The level of support experienced by patients in various social situations was determined using the Multidimensional Scale of Perceived Social Support (MSPSS) [13]. The scale has three dimensions and twelve items, ranging from ‘strongly disagree’ to ‘strongly agree’. The scores are graded on a 1–7 scale, with low to high scores representing views of social support ranging from weak to robust. The scale has an overall Cronbach’s α coefficient of 0.899, indicating good reliability.

#### Peripheral Blood Index Determination

Three milliliters of cubital venous blood were collected from each patient and centrifuged for 8 min at 2500 rpm and a radius of 10 cm. The serum was collected and stored at –70 °C until testing. Serum SCC-Ag levels were determined using electrochemiluminescence.

### Survey Methodology

The patients’ depression scores were obtained from the hospital medical record system 3–7 days after surgery (T1), 1–3 days after the first chemotherapy (T2), 1–3 days after the fourth chemotherapy (T3), 1–3 days after the end of chemotherapy (T4) and 3 months later (T5). Two researchers independently extracted and cross-checked the data to minimise errors (Cohen’s κ = 1).

### Statistical Methods

The data were examined and processed using SPSS 26.0 (IBM Corp., Armonk, NY, USA), Mplus 8.3 (Muthén & Muthén, 1998–2017, Los Angeles, CA, USA) and Python 3.11. The Shapiro–Wilk test was used to determine normality, and measured data that passed the test were expressed as ($\bar{x}$ ± s). Multiple-group comparisons were performed using one-way ANOVA. Measurement data were reported as medians (interquartile ranges), and the Kruskal–Wallis H test was used to compare several groups. Count data was presented as [n (%)], and intergroup comparisons were made using the χ^2^ test. This study used the LGMM to examine depression trajectories during CCRT for cervical cancer patients. The Akaike information criterion (AIC), Bayesian information criterion (BIC), sample-adjusted BIC (aBIC) and information entropy are used as model evaluation indicators. Logistic regression was performed to identify contributing factors, with *p* < 0.05 indicating statistical significance.

## Results

### Comparison of Clinical Data Between the Two Groups of Patients

No significant differences between the groups were found in terms of age, BMI, educational level, marital status, menopause, HPV infection, lymph node metastases or SCC-Ag levels (*p* > 0.05). The depressed group had higher rates of household monthly income per capita of less than 5000 RMB (1 USD = 7.1 RMB), stage III/IV tumour stage and avoidance/submission coping methods than the non-depressed group (*p* < 0.05) and had lower MSPSS scores (*p* < 0.05, Cohen’s *d* = –0.600; Table 1).

**Table 1.** **Comparison of clinical data of the two groups of patients**.

Observation indicators		Depressed group (n = 114)	Non-depressed group (n = 46)	*t*/$\chi^{2}$/*Z* value	*p*-value
Age		47.08 ± 6.93	48.28 ± 5.29	−1.204	0.231
BMI				0.810	0.368
	<24 kg/m^2^	84 (73.68)	37 (80.43)		
	≥24 kg/m^2^	30 (26.32)	9 (19.57)		
Educational level				2.740	0.098
	Middle school and below	66 (57.89)	20 (43.48)		
	High school and above	48 (42.11)	26 (56.52)		
Marital status				-	1.000
	Married	107 (93.86)	44 (95.65)		
	Single/Divorced/Widowed	7 (6.14)	2 (4.35)		
Average monthly household income				10.595	0.001
	<5000 RMB (1 USD = 7.1 RMB)	89 (78.07)	24 (52.17)		
	≥5000 RMB (1 USD = 7.1 RMB)	25 (21.93)	22 (47.83)		
Menopause				0.035	0.852
	Yes	81 (71.05)	32 (69.57)		
	No	33 (28.95)	14 (30.43)		
HPV infection				2.340	0.126
	Have	36 (31.58)	9 (19.57)		
	None	78 (68.42)	37 (80.43)		
Tumour staging				4.714	0.030
	Stage I/II	87 (76.32)	42 (91.30)		
	Stage III/IV	27 (23.68)	4 (8.70)		
Lymph node metastasis				3.054	0.081
	Have	41 (35.96)	10 (21.74)		
	None	73 (64.04)	36 (78.26)		
Coping methods				32.678	<0.001
	Face	23 (20.18)	31 (67.39)		
	Avoid/surrender	91 (79.82)	15 (32.61)		
MSPSS score		61.47 ± 11.27	68.33 ± 11.85	−3.431	0.001
SCC-Ag (ng/mL)		6.13 ± 0.72	6.09 ± 0.69	0.293	0.770
Depression score		12 (10, 14.5)	4 (3, 5)	−9.961	<0.001

Note: BMI, Body Mass Index; SCC-Ag, Squamous Cell Carcinoma Antigen; MSPSS, Multidimensional Scale of Perceived Social Support; HPV,Human Papilloma Virus.

### Identification and Determination of Depression Trajectory Types in Patients With Cervical Cancer

A latent variable growth mixture model was used to fit latent classes of the patients’ depressive trajectory. The fit indices for each class are presented in Table 2. As the number of model classes increases, the AIC, BIC and aBIC values decrease, whereas entropy increases. When the number of classes reached 4, the AIC, BIC and aBIC values increased, but the entropy remained low. After careful deliberation, three latent class models were selected.

**Table 2.** **Model fitting results of depression in patients with cervical cancer (n = 114)**.

G	Loglik	conv	npm	AIC	BIC	aBIC	entropy	Class probability
1	−2420.191	1	8	4862.575	4960.597	5044.612	-	1
2	−2396.411	1	16	4795.694	4724.583	4894.752	0.946	0.544/0.456
3	−2287.152	1	24	4218.272	4259.492	4388.385	0.984	0.272/0.474/0.254
4	−2351.687	1	32	4427.043	4385.723	4458.154	0.961	0.237/0.246/0.298/0.219

Note: AIC, Akaike information criterion; BIC, Bayesian information criterion; aBIC, sample-adjusted BIC.

### Naming of Depression Trajectory Categories in Patients with Cervical Cancer

**Fig. 2.**
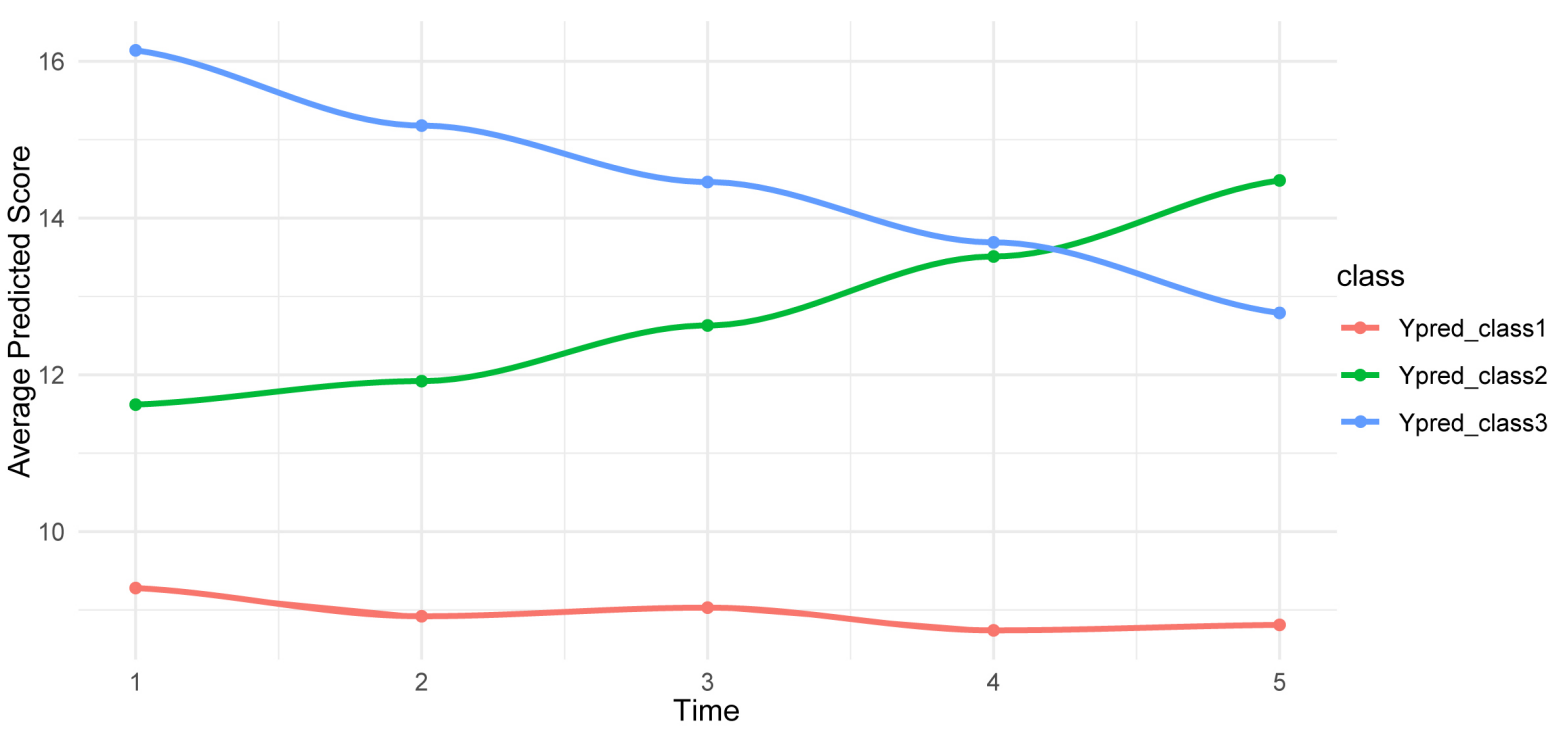
**Development trends of three trajectories of depression in patients with cervical cancer**. Class 1: low-level stable group; class 2: medium-level rising group; class 3: high-level declining group.

The subgroup trajectories were shown using time points as the horizontal axis and depression scores as the vertical axis. There were 31 cases classified as low-level steady, 54 as moderately rising and 29 as high-level decreasing. Fig. 2 shows the developmental trajectories of each category.

### Comparison of the Development Trajectory of Depression in Patients with Cervical Cancer

In cervical cancer patients, univariate analysis revealed significant differences in per capita monthly household income, tumour stage, coping style and MSPSS score (*p* < 0.05, η^2^ = 0.837) among the three latent categories of depression developmental trajectories. See Table 3.

**Table 3.** **Univariate analysis of depression trajectory categories in cervical cancer patients (n = 114)**.

Observation indicators		N	Low level stable (n = 31)	Moderate level increase (n = 54)	High level decline (n = 29)	*F*/$\chi^{2}$ value	*p*-value
Age		114	47.29 ± 6.87	46.65 ± 6.95	47.59 ± 7.14	0.193	0.824
BMI						0.282	0.868
	<24 kg/m^2^	84	22 (70.97)	41 (75.93)	21 (72.41)		
	≥24 kg/m^2^	30	9 (29.03)	13 (24.07)	8 (27.59)		
Educational level						1.198	0.549
	Middle school and below	66	16 (51.61)	31 (57.41)	19 (65.52)		
	High school and above	48	15 (48.39)	23 (42.59)	10 (34.48)		
Marital status						0.649	0.783
	Married	107	30 (96.77)	50 (92.59)	27 (93.10)		
	Single/Divorced/Widowed	7	1 (3.23)	4 (7.41)	2 (6.90)		
Average monthly household income						7.067	0.029
	<5000 RMB (1 USD = 7.1 RMB)	89	19 (61.29)	46 (85.19)	24 (82.76)		
	≥5000 RMB (1 USD = 7.1 RMB)	25	12 (38.71)	8 (14.81)	5 (17.24)		
Menopause						0.466	0.792
	Yes	81	21 (67.74)	40 (74.07)	20 (68.97)		
	No	33	10 (32.26)	14 (25.93)	9 (31.03)		
HPV infection						0.807	0.668
	Have	36	8 (25.81)	19 (35.19)	9 (31.03)		
	None	78	23 (74.19)	35 (64.81)	20 (68.97)		
Tumour staging						7.465	0.024
	Stage I/II	87	29 (93.55)	39 (72.22)	19 (65.52)		
	Stage III/IV	27	2 (6.45)	15 (27.78)	10 (34.48)		
Lymph node metastasis						0.896	0.639
	Have	41	9 (29.03)	21 (38.89)	11 (37.93)		
	None	73	22 (70.97)	33 (61.11)	18 (62.07)		
Coping methods						9.090	0.011
	Face	23	12 (38.71)	7 (12.96)	4 (13.79)		
	Avoid/surrender	91	19 (61.29)	47 (87.04)	25 (86.21)		
MSPSS score		114	67.94 ± 10.40	60.96 ± 10.39	55.52 ± 10.41	10.806	<0.001
SCC-Ag (ng/mL)		114	6.18 ± 0.74	6.10 ± 0.78	6.13 ± 0.59	0.134	0.875

Note: BMI, Body Mass Index; SCC-Ag, Squamous Cell Carcinoma Antigen; MSPSS, Multidimensional Scale of Perceived Social Support; HPV, Human Papilloma Virus.

### Analysis of Influencing Factors of Depression Development Trajectory in Patients With Cervical Cancer

Logistic regression analysis was performed using statistically significant variables in the univariate analysis as independent variables (average monthly household income <5000 RMB = 1, ≥5000 RMB = 0; tumour staging III/IV = 1, I/II = 0; coping methods avoidance/submission = 1, confrontation = 0; Original MSPSS score input), the depressive mood subgroup as the dependent variable and the low-level stable group as the reference group. Patients with a monthly family income of less than 5000 RMB, tumour stage III/IV, avoidance/submission coping style and lower MSPSS scores were more likely to enter the medium-level rising and high-level declining groups (*p* < 0.05; Table 4).

**Table 4.** **Analysis of factors affecting the development trajectory of depression in patients with cervical cancer**.

Group	Variable	*β*	*SE*	*Wald*	*p*	*OR*	*95% CI*
Moderate level Rising group vs. low level stable group	Average monthly household income	0.340	0.142	5.732	0.017	1.405	1.306–1.862
	Tumour staging	0.268	0.141	3.613	0.043	1.308	1.016–1.748
	Coping methods	0.517	0.219	5.573	0.018	1.677	1.296–1.879
	MSPSS score	−0.083	0.029	8.191	0.005	0.920	0.869–0.975
High level Decline group vs. low level Stable group	Average monthly household income	0.202	0.091	4.927	0.028	1.224	1.035–1.652
	Tumour staging	0.139	0.074	3.528	0.047	1.149	1.021–1.475
	Coping methods	0.419	0.181	5.359	0.021	1.521	1.432–1.703
	MSPSS score	−0.139	0.043	10.449	0.001	0.870	0.799–0.946

Note: MSPSS, Multidimensional Scale of Perceived Social Support; SE, Standard Error; OR, Odds Ratio; CI, Confidence Interval.

## Discussion

### Heterogeneity in Depression Trajectories in Patients Undergoing CCRT for Cervical Cancer

Most patients with cervical cancer experience negative emotions during CCRT, and depression is the most prevalent [14]. Depression not only might worsen a patient’s quality of life but also can hinder chemoradiotherapy, worsening the prognosis [15]. This study used a latent variable growth mixed model to examine the progression of depression in cervical cancer patients undergoing CCRT, aiming to provide personalised intervention strategies for depression management and identify different depression trajectories.

### Depression Trajectory Classification, Influencing Factors and Implications for Personalised Intervention in Cervical Cancer Patients Undergoing Radiotherapy and Chemotherapy

In this study, the development trajectory of depression in patients with cervical cancer was divided into three types: low-level stable, medium-level rising and high-level declining groups. The level of depression in these patients varies by group. Different screening procedures may be used to control the progression of depression in patients with various types of depression trajectories. Patients in the low-level stable group have a low threshold of depression and may have natural protective factors, and thus screening should focus on maintaining stability rather than active intervention. Patients in the medium-level rising group show a gradual increase in depression, which may be related to the accumulation of side effects of treatment and fear of disease progression. Thus, high-frequency screening is needed to capture the inflection point of deterioration. Patients in the high-level decline group have high baseline depression levels but exhibit a trend toward remission. This trend may be influenced by interventions or their own adjustment ability, and screening should consider treatment consolidation and relapse warning. Further analysis of this study revealed that patients with a family monthly income of less than 5000 RMB, tumour stage III/IV, avoidance/submission coping style and lower MSPSS scores were more likely to enter the medium-level rising and high-level declining groups than the low-level stable group. Cervical cancer patients frequently require multi-stage and time-consuming radiotherapy and chemotherapy, which places a significant financial burden on patients and their families, particularly those from low-income families. They are subjected to increased economic pressure, which exacerbates depression to some extent [16]. The higher the pathological stage of cervical cancer, the more severe the disease and the more likely complications occur, and the degree of depression tends to increase. This primary reason is that cancer causes varying degrees of damage to the patient’s bodily function, using a large amount of body energy and exacerbating the patient’s depression [17]. Previous survey data on cervical cancer patients revealed that when confronted with their own sickness, patients who adopted a coping style of facing the disease experienced lower levels of depression than those who chose to give in or face the coping style [18]. The findings of this investigation have once again confirmed this. Previous research [19] found that good social support helps minimise depression in patients receiving cervical cancer radiotherapy and chemotherapy, which is consistent with this study. This suggests that in clinical work, health education manuals or propaganda can be used to popularise disease-related knowledge for patients, assist patients in correcting misconceptions, such as ‘disease stigmatisation’ and ‘self-denial’, and promote it from multiple levels and subjects, combining clinical practice with family care integration to improve patients’ MSPSS scores and reduce the risk of depression. As a result, patients who meet the criteria listed above can be evaluated for depression early and given priority in psychological care programs. A team of oncologists, psychotherapists and social workers can be formed to develop individualised programs for patients with varied trajectories to control the progression of depression [20]. However, the study had certain drawbacks. The sample size is small, and it comes from a single medical centre, which may affect the universality of trajectory classification. In the future, multi-centre research will be conducted to increase the sample size and lengthen the follow-up period to ensure the stability of trajectory categorisation.

## Conclusions

Depression in patients with cervical cancer follows three different developmental trajectories. Household monthly income, tumour stage, coping style and MSPSS score are potential influencing factors of these trajectories. Early intervention targeting these potential influencing factors is necessary to control the progression of depression.

## Availability of Data and Materials

This study’s data were derived from 160 cervical cancer patients who underwent concurrent chemoradiotherapy at our hospital between July 2023 and June 2025. The data presented in this study are available on request from corresponding author. The data are not publicly available due to privacy.
